# Trichoblastoma in a 10-year-old female

**DOI:** 10.1016/j.jdcr.2026.09.002

**Published:** 2026-09-08

**Authors:** Aimal Fatima, Duncan Lambie, Genevieve Casey

**Affiliations:** a Casey Dermatology, Brisbane City, QLD, Australia; b Sullivan Nicolaides Pathology, Brisbane, QLD, Australia; c Frazer Institute, University of Queensland, Brisbane, QLD, Australia; d Pathology Queensland, Princess Alexandra Hospital, Brisbane, QLD, Australia

**Keywords:** adnexal tumors, basal cell carcinoma, case reports (general dermatology), clinical cases, dermoscopy, pediatrics, tumors/neoplasm, trichoblastoma, trichoepithelioma

## Introduction

Trichoblastoma is a rare, benign, adnexal tumor derived from follicular germinative cells that occurs predominantly in adults, with limited pediatric cases reported in the literature. Clinically, a trichoblastoma typically presents as a solitary, slow-growing, skin-colored papule or nodule on the face or scalp, mimicking a basal cell carcinoma (BCC) both clinically and dermoscopically. Histopathological examination is essential to distinguish between the two, as trichoblastoma demonstrates variable stromal condensation and pilar differentiation, in contrast to a BCC, which typically shows basaloid nodules with marked peripheral palisading and cleft formation between tumor islands and adjacent stroma.^1^ The immunohistochemical marker, pleckstrin homology-like domain family A member 1, also known as PHLDA1 (T-cell death-associated gene 51), is particularly helpful in distinguishing between trichoblastoma (TB) and BCC. Complete surgical excision is recommended due to the rare risk of malignant transformation.

We report a case of trichoblastoma in a 10-year-old female presenting as a solitary facial nodule, highlighting the rarity of this entity in the pediatric population and the need for histopathological examination to exclude basal cell carcinoma as a differential diagnosis.

## Case report

A 10-year-old female presented with a 2-year history of a slowly enlarging solitary nodule on the right medial cheek. She was initially seen by a dermatologist and plastic surgeon in Korea; however, no definitive diagnosis was made. A bleomycin injection (0.5 mL) was administered by a plastic surgeon 3 months before her clinic presentation, resulting in temporary flattening of the lesion. Following this injection, the lesion became pruritic, crusted, and partially desquamated, leaving an area of hypopigmentation.

On examination, a firm, well-circumscribed, 4 × 5 mm skin-colored dermal nodule was present on the right medial cheek (Fig 1). Dermoscopy demonstrated white pearly areas with branching vessels. No features suggestive of an associated syndrome were identified. Differential diagnoses included pilomatrixoma and a hypertrophic scar. A referral to a plastic surgeon was made for an excisional biopsy.

**Fig 1:**
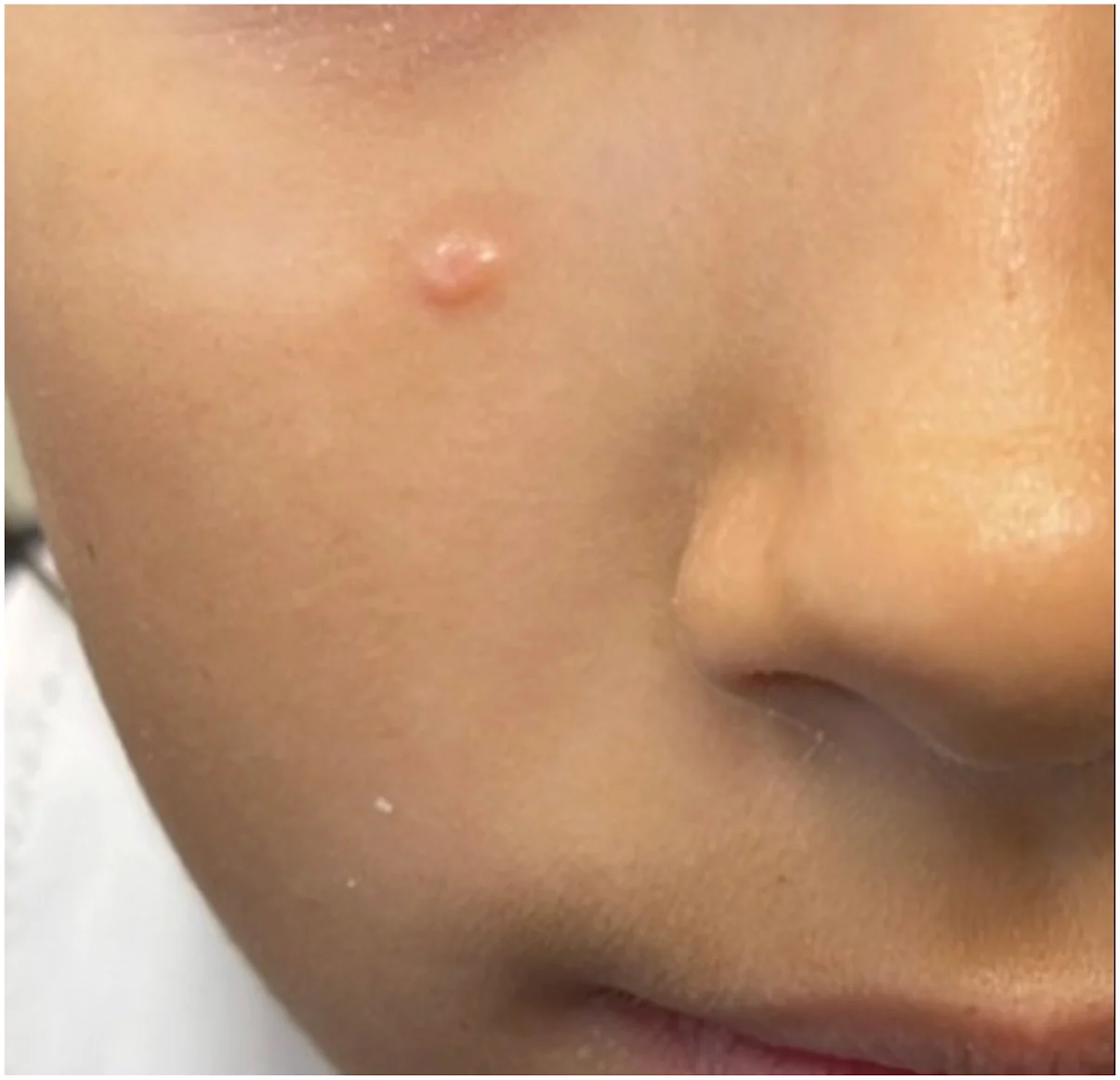
A firm, well-circumscribed, 4 × 5 mm cream-colored dermal nodule on the patient’s right medial cheek.

Histology showed a circumscribed nodular lesion composed of well-defined basaloid islands, with solid and cribriform architecture, cellular fibrous stroma and a few papillary mesenchymal bodies at the edges of some nests, consistent with a benign trichoblastoma (Figs 2 and 3). The morphological features were considered diagnostic, and immunohistochemistry, including PHLDA1, was not performed. There were no features on histology to suggest that the intralesional bleomycin had altered the histologic structure of the trichoblastoma. The lesion was completely excised with clear margins.

**Fig 2:**
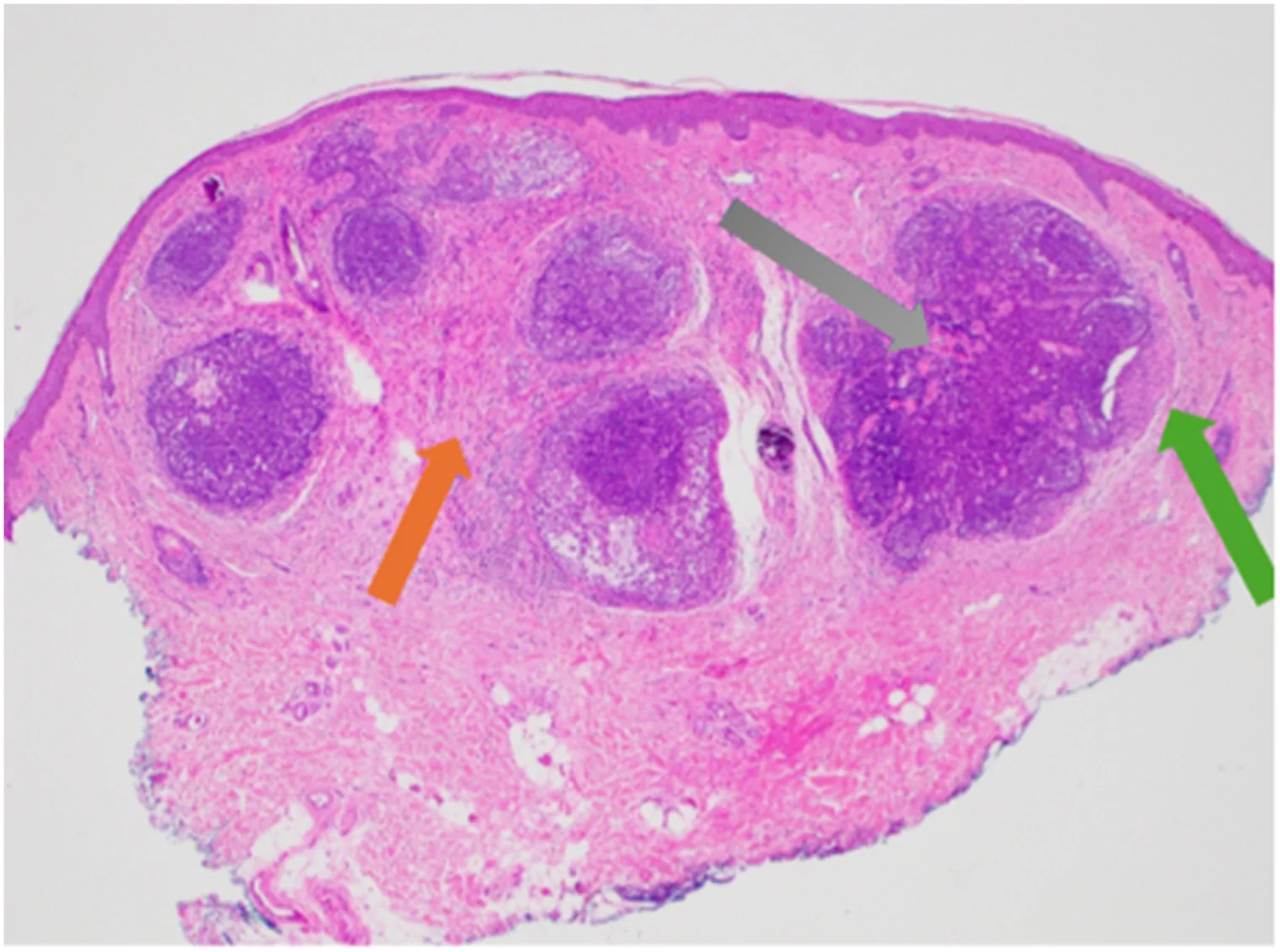
Dermal tumor composed of islands of basaloid cells with solid and cribriform architecture (*gray arrow*), cellular fibrous stroma (*orange arrow*) and papillary mesenchymal bodies (*green arrow*). Hematoxylin & Eosin 40× magnification.

**Fig 3:**
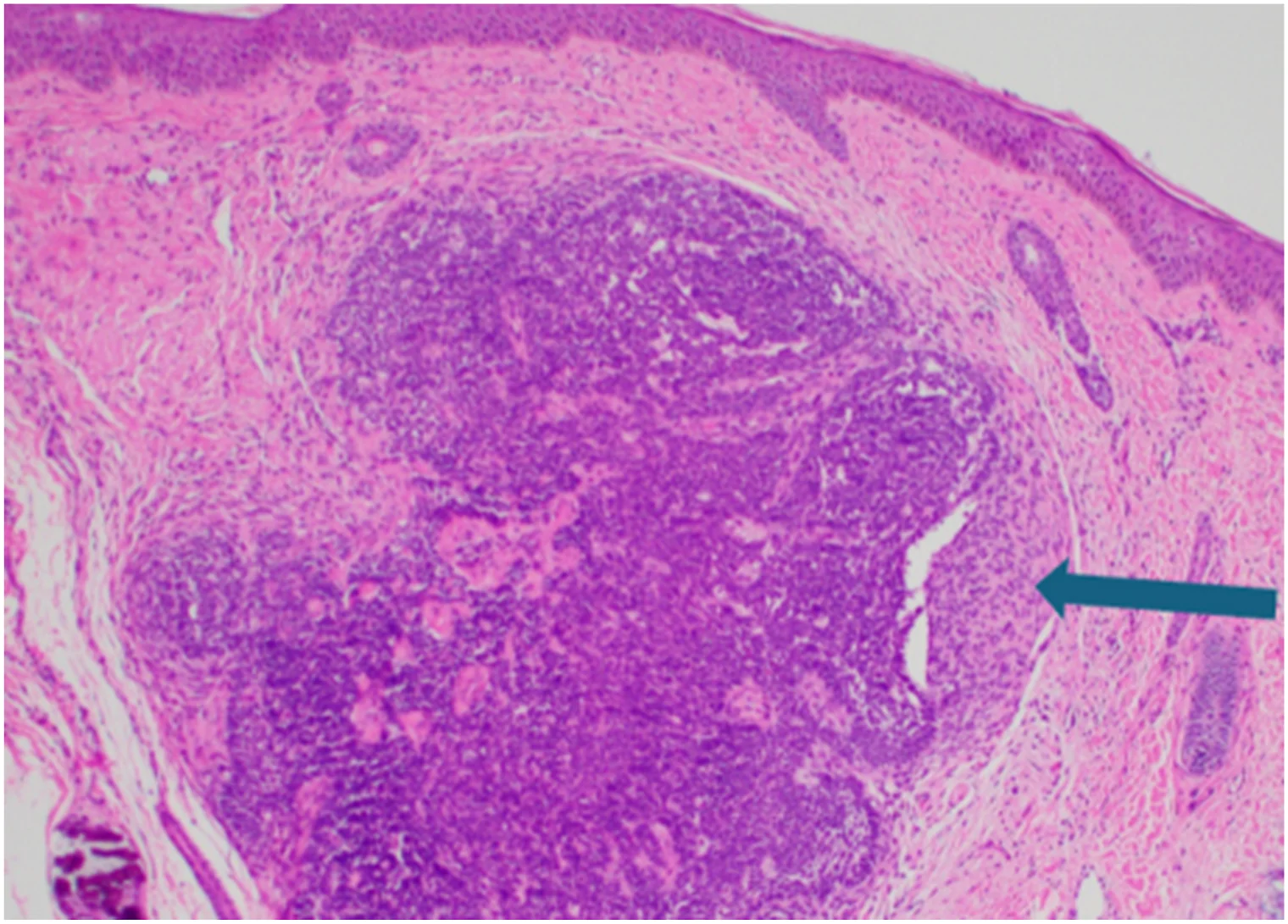
Solid and cribriform tumor lobule with condensed cellular fibrous stroma representing papillary mesenchymal body (*arrow*). Hematoxylin & Eosin 100× magnification.

The family was reassured and follow-up was arranged to ensure wound healing and scar maturation; at 12-month follow-up, the wound had healed well with no evidence of recurrence (Fig 4).

**Fig 4:**
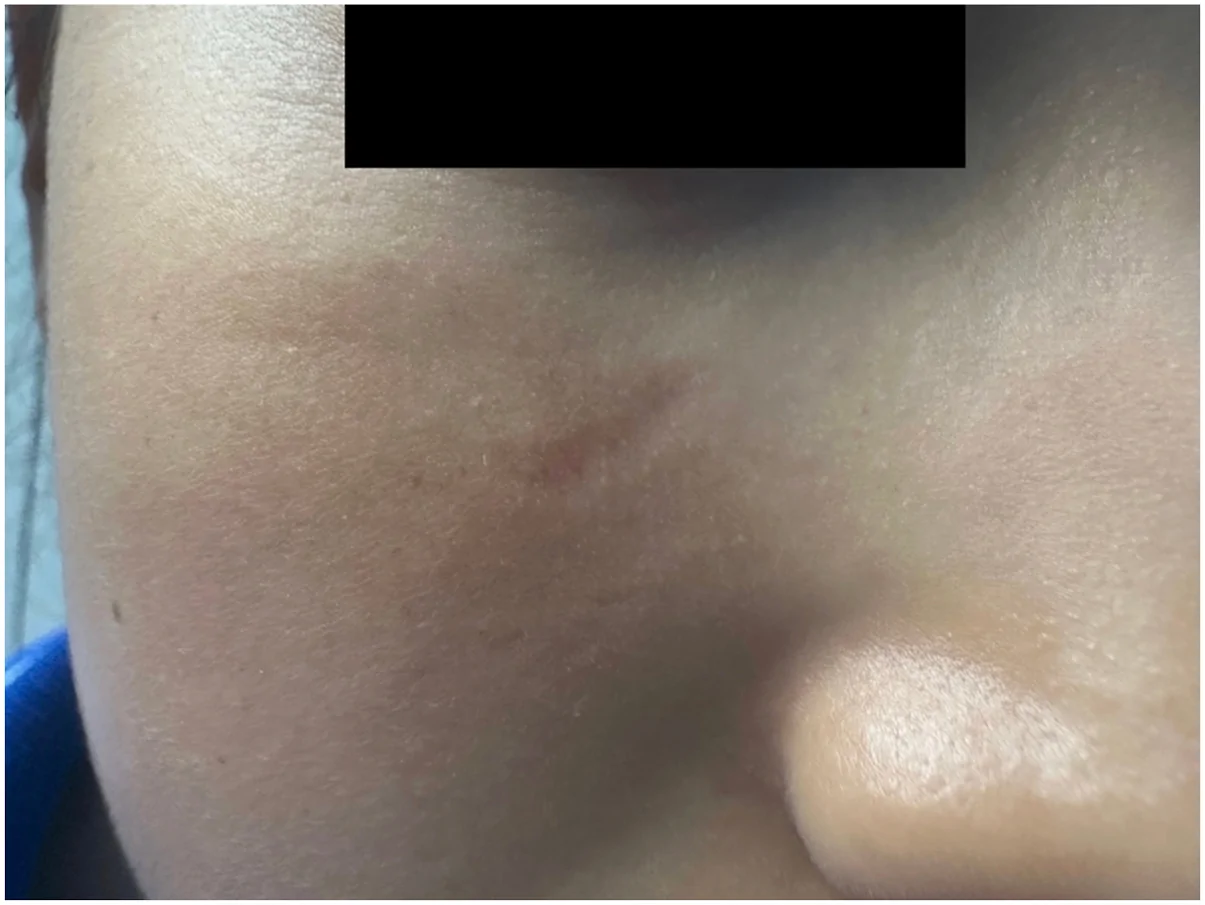
Right cheek at 12 months postexcision, demonstrating a well-healed surgical scar with no clinical evidence of recurrence.

## Discussion

TB is a rare, benign, adnexal tumor derived from follicular germinative cells.^1^ It typically presents as a solitary 0.2-1 cm skin-colored, well-circumscribed, slow-growing papule or nodule, usually on the face or scalp, though less commonly may present on the trunk, proximal extremities, and perianal areas.^1^

Trichoblastomas are extremely rare in childhood, occurring predominantly in adults aged 40-50 years.^1^ Our patient adds to the literature on trichoblastoma in children. Case reports include a primary trichoblastoma in a 10-day-old male on the right submandibular region,^2^ 11-and 19-year-old females with a primary trichoblastoma on the right cheek and right scalp,^3^^,^^4^ respectively, and a 17-year-old male with four trichoblastomas on the forehead, bilateral posterior scalp, and neck.^5^ There are also reports of a trichoblastoma in a 15-year-old female on the parietal scalp,^6^ and a 4-year-old male on the left cheek, though both were secondary tumors arising from a naevus sebaceous.^7^

An important differential diagnosis of a trichoblastoma is basal cell carcinoma. Histologically, trichoblastomas are usually well-circumscribed, non-ulcerated neoplasms that are confined to the dermis, and rarely invade the subcutaneous tissue.^8^ A trichoblastoma can be distinguished from a BCC by its variable stromal condensation and pilar differentiation.^1^ In contrast, BCC is a basaloid neoplasm that arises from the epidermis, typically showing basaloid nodules with marked peripheral palisading and cleft formation between tumor islands and adjacent stroma.^1^ PHLDA1 (T-cell death-associated gene 51), a follicular stem cell marker, has been proposed as the best immunohistochemical marker for distinguishing trichoblastoma from BCC, given it is characteristically positive in TB and negative in BCC.^8^ In basaloid neoplasms arising within a naevus sebaceous, the negative results for PHLDA1 support a diagnosis of BCC rather than TB.^8^ Ductal structures typically stain positively for epithelial membrane antigen and carcinoembryonic antigen.^8^ In our case, the combination of well-defined basaloid islands with solid and cribriform architecture, a cellular fibrous stroma and papillary mesenchymal bodies was sufficiently characteristic to establish the diagnosis, and immunohistochemistry was not required.

Another differential diagnosis included pilomatrixoma, a benign tumor that originates from the hair follicle matrix and typically presents as a solitary, asymptomatic, firm nodule. The absence of ghost cells, calcification and ossification on our patient’s histopathology excluded this as a diagnosis.

Clinically and dermoscopically, trichoblastoma may mimic BCC, making histopathological examination essential to confirm the diagnosis and exclude malignant mimickers. Although malignant transformation is exceedingly rare, it has been reported in the literature and may be suggested by rapid enlargement or spontaneous bleeding; complete excision is therefore recommended.^9^ This case highlights the importance of considering trichoblastoma in the differential diagnosis of persistent facial nodules in children.

## Conflicts of interest

None disclosed.
